# Successful Removal of Uterus and Both Ovaries by Abdominal Section

**Published:** 1866-01

**Authors:** 


Successful Removal of Uterus and both Ovaries by Abdominal Section.—Dr. Horatio R. Storer of Boston, sends us a pamphlet containing a detailed account of the operation for removal of the uterus and ovaries, which he made successfully upon an unmarried lady, aged 47, in September last. The pamphlet also contains a very complete account of the literature of this operation, which must prove very interesting to all those who propose repeating it. We most heartily congratulate Dr. Storer upon his success, for perhaps no operation in surgery would require more moral courage to perform, than this, since no one has been more uniformly attended by fatal results. The paper is also published in *American Journal of Medical Sciences* for Januury, 1866. Thus many of our readers will see the paper entire. The author seems sanguine of success in such operations, and gives reasons for his conclusions which appear quite satisfactory.

